# Elevated Carcinoembryonic Antigen Levels Predict Failure to Reach Surgery in Patients with Borderline Resectable Pancreatic Cancer Referred to Neoadjuvant Therapy

**DOI:** 10.1245/s10434-025-17433-3

**Published:** 2025-05-13

**Authors:** Arielle Jacover, Tamar Beller, Nedaa Mahamid, Noa Avishay, Karny Ilan, Yoav Elizur, Havi Murad, Ron Pery, Rony Eshkenazy, Yuri Goldes, Talia Golan, Ido Nachmany, Niv Pencovich

**Affiliations:** 1 Department of General Surgery and Transplantation, Faculty of Medicine and Health Sciences, Sheba Medical Center, Tel-Hashomer, Tel-Aviv University, Tel-Aviv, Israel; 2 Department of Oncology, Faculty of Medicine and Health Sciences, Sheba Medical Center, Tel-Hashomer, Tel-Aviv University, Tel-Aviv, Israel; 3 Department of Internal Medicine, Faculty of Medicine and Health Sciences, Ward ‘B’, Sheba Medical Center, Tel-Hashomer, Tel-Aviv University, Tel-Aviv, Israel; 4 Biostatistics and Biomathematics Unit, Gertner Institute, Sheba Medical Center, Tel-Hashomer, Ramat-Gan, Israel

**Keywords:** CEA, Chemotherapy, Marker, Tumor, Radiation therapy, Locally advanced

## Abstract

**Introduction:**

Neoadjuvant therapy (NT) is generally preferred over upfront surgery for borderline resectable pancreatic ductal adenocarcinoma (BR-PDAC), but many patients fail to reach surgical resection. This study evaluates real-world outcomes of NT in BR-PDAC and identifies predictors of failure to proceed to surgery.

**Methods:**

A retrospective analysis of patients with resectable and BR-PDAC diagnosed between January 2015 and July 2024 was performed. Patient and disease characteristics were assessed to identify factors associated with NT dropout and failure to achieve surgical resection.

**Results:**

Of 161 BR-PDAC patients, 111 (69%) were referred to NT and 50 (31%) underwent upfront surgery. Among those referred to NT, 78 (70%) completed therapy and underwent resection. Reasons for failure to reach surgery included local tumor progression (39%), newly developed metastases (18%), and intraoperative findings (27%). Patients failing to reach surgery had significantly higher baseline bilirubin, white blood cell count, and carcinoembryonic antigen (CEA) levels. Elevated CEA significantly predicted surgical failure (adjusted odds ratio: 0.68 per 5-unit increase). Local progression was the primary cause of surgical failure in patients with elevated CEA (60%). Patients achieving resection had significantly improved overall survival (OS). There was no significant difference in OS or disease-free survival (DFS) between patients undergoing upfront surgery and those completing NT followed by resection.

**Conclusions:**

Elevated baseline CEA predicts failure to achieve surgical resection after NT, primarily owing to local progression. Multicenter studies are essential to refine patient selection criteria for upfront surgery and optimize personalized therapeutic strategies.

**Supplementary Information:**

The online version contains supplementary material available at 10.1245/s10434-025-17433-3.

Pancreatic ductal adenocarcinoma (PDAC) remains one of the most lethal malignancies, characterized by poor prognosis due to late-stage diagnosis and aggressive disease progression.^1^ Borderline resectable PDAC (BR-PDAC) represents a distinct subset of PDAC, characterized by anatomical criteria, biological factors, and patient functional status.^2^ These factors collectively predict the need for technically challenging surgeries, often involving vascular resection and reconstruction, aggressive tumor biology that may render surgical intervention futile, and patient conditions that may impair recovery from surgical complications, respectively. Current treatment paradigms for BR-PDAC increasingly favor neoadjuvant therapy (NT)—chemotherapy or chemoradiotherapy—to downstage tumors, improve the likelihood of achieving clear surgical margins, and provide a “test of time” to assess disease biology and rehabilitate patients.^3^ However, the optimal treatment strategy for BR-PDAC remains a subject of ongoing debate,^4^ and additional parameters for differential patient selection have a high clinical demand.

Among NT regimens, FOLFIRINOX has emerged as a widely used option, demonstrating superior response rates compared with gemcitabine-based treatments, particularly in patients with BR-PDAC.^5,6^ However, while evidence suggests that NT followed by surgery and adjuvant therapy can benefit patients with BR-PDAC, challenges such as early dropout due to treatment toxicity or disease progression complicate clinical decision-making.^5^ Additionally, NT often necessitates invasive procedures, such as endoscopic ultrasound (EUS)-guided biopsy and common bile duct (CBD) stenting, which can introduce complications that delay or even preclude further treatment.

Although recent studies have provided insights into clinical and radiological predictors of treatment response and survival in BR-PDAC,^7,8^ significant gaps remain in understanding the factors associated with dropout from NT. Identifying these factors is crucial to optimize treatment strategies and guide the decision-making process between NT and upfront surgery in patients with BR-PDAC. This study aims to identify the factors that predict failure to proceed to surgery following NT in patients with BR-PDAC and to assess the impact of this result on long-term outcomes. For a comprehensive analysis, patients with BR-PDAC who underwent upfront surgery and those with resectable PDAC were included as control groups.

## Methods

This retrospective study included patients with BR-PDAC treated at a tertiary referral center between January 2015 and July 2024. All cases were reviewed during weekly multidisciplinary team (MDT) meetings comprising pancreatic cancer oncologists, radiation oncologists, radiologists, hepatopancreatobiliary surgeons, upper gastrointestinal surgeons, and gastroenterologists. Data were retrospectively extracted from electronic medical records maintained by the surgery and oncology departments using a novel MDClone^©^ software, a data extraction and synthesis platform connected to our facility’s medical records system (http://www.mdclone.com).

Collected data included patient demographics, medical history, comorbidities, tumor characteristics on imaging, details of NT, perioperative laboratory indices, surgical pathology, postoperative complications, adjuvant treatments, disease recurrence, and survival outcomes. All extracted data were manually reviewed and validated. Noncoded information, including complications during NT, reasons for NT discontinuation, presenting symptoms, MDT decisions, and additional clinical details, were manually retrieved from patient medical charts. Postoperative complications were classified according to the Clavien–Dindo (CD) system, with major complications defined as CD grade > 3a.^9^ ChatGPT was used to improve grammar and context.

All study procedures adhered to the principles of the Declaration of Helsinki. The institutional review board at Sheba Medical Center approved the study (approval reference no. SMC-9498-22), and the ethics committee waived the requirement for informed consent owing to the retrospective nature of the research.

### Statistical Analysis

Differences between groups were compared using the nonparametric Wilcoxon rank sum test for continuous variables, Fisher’s exact test, and chi-squared test for categorical variables. Patients with missing data values were excluded from the calculation of medians/percentages and statistical comparison for the respective variable. Predictors of disease recurrence and survival in patients with BR-PDAC referred for surgery were assessed using a multivariate logistic regression model. The analysis was performed using the “Enter” method, incorporating variables selected on the basis of clinical rationale. All *p* values are two-tailed, and the alternative hypothesis was considered significant if *p *≤ 0.05. Missing data were omitted from the analysis without imputations. Candidate variables to be predictors for failure to reach surgical resection were identified using a univariate logistic regression model (*p *< 0.1). Multivariable logistic regression was conducted using the stepwise method. Only those significant at the 5% level remained in the final model. Missing data were omitted from the analysis without imputations. Survival analysis was applied using Cox (proportional hazard) regression analysis, and the statistical significance of Kaplan–Meyer curves is reported using the log-rank test. Patients who died within 90 days of surgery were excluded from the survival analysis. All statistical analyses were done using SAS software, version 9.4 for UNIX, and R studio version 3.6.2.

## Results

Between January 2015 and July 2024, a total of 166 patients were diagnosed with BR-PDAC. Of these, 111 patients (69%) were referred for NT while 50 patients (29%) were directed to upfront surgery. An additional five patients were referred to palliative radiation or hospice care owing to poor overall health status or personal preference and were excluded from the analysis. Patient demographics, medical history, and disease presentation are detailed in Table 1. The classification of tumors as borderline resectable was based on anatomical, biological, and functional status criteria.^2^ Among the 161 patients, 57 (35%) were classified as having BR-PDAC on the basis of anatomical criteria alone, 68 (42%) on the basis of biological criteria alone, and 30 (19%) on the basis of both anatomical and biological criteria. An additional five patients (3%) were classified as having BR-PDAC owing to functional status (Table 1). In one patient, the rationale for classifying the tumor as BR-PDAC was unavailable. Patients referred for upfront surgery were generally older and more frequently presented with symptoms such as pruritus and jaundice. At the time of diagnosis, these patients also exhibited higher bilirubin and CA 19-9 levels (Table 1). Notably, 75% of patients referred to NT were classified as having BR-PDAC on the basis of anatomical factors, compared with only 6% of those referred to upfront surgery. In contrast, the majority of patients in our cohort who underwent upfront surgery were classified as having BR-PDAC owing to biological factors, such as elevated CA 19-9 levels or imaging evidence of peripancreatic lymph node involvement.

**Table 1:** Characteristics of patients with borderline PDAC who were referred to neoadjuvant treatment followed by surgery, or upfront surgery

Characteristic	All, *N* = 161^1^	Referred to NT, *N* = 111^1^	Referred to upfront surgery, *N* = 50^1^	*p*-value^2^
*Demographics and medical history*				
Age (years)	68 (62, 74)	67 (60, 73)	71 (63, 76)	0.027
Gender female	68 (43%)	48 (44%)	20 (40%)	0.6
Active smoker	53 (33%)	40 (37%)	13 (26%)	0.2
Diabetes	80 (50%)	54 (50%)	26 (52%)	0.8
HTN	69 (43%)	44 (40%)	25 (50%)	0.3
IHD	19 (12%)	16 (15%)	3 (6.0%)	0.12
COPD	1 (0.6%)	1 (0.9%)	0 (0%)	> 0.9
Asthma	10 (6.3%)	5 (4.6%)	5 (10%)	0.3
CVA	7 (4.4%)	4 (3.7%)	3 (6.0%)	0.7
CRF	4 (2.5%)	3 (2.8%)	1 (2.0%)	> 0.9
*Presenting symptoms*				
Pruritus	11 (7.0%)	4 (3.7%)	7 (14%)	0.038
Abdominal pain	73 (46%)	53 (50%)	20 (40%)	0.3
Weight loss	69 (44%)	48 (45%)	21 (42%)	0.7
Back pain	18 (11%)	13 (12%)	5 (10%)	0.7
Jaundice	68 (43%)	40 (37%)	28 (56%)	0.028
No symptoms	14 (8.9%)	7 (6.5%)	7 (14%)	0.14
Other symptoms	28 (18%)	20 (19%)	8 (16%)	0.7
CBD stenting	68 (45%)	47 (46%)	21 (43%)	0.7
*Relevant laboratory indices at disease presentation*				
CA19-9	636 (129, 1,514)	312 (84, 1,306)	1,157 (655, 1,616)	<0.001
CEA	4 (2, 7)	3 (2, 7)	4 (2, 6)	> 0.9
Albumin	3.80 (3.50, 4.10)	3.80 (3.50, 4.10)	3.80 (3.40, 4.00)	0.8
Bilirubin	0.6 (0.4, 3.2)	0.5 (0.4, 1.0)	3.4 (0.8, 8.3)	<0.001
WBC	7.00 (5.70, 9.00)	6.93 (5.63, 9.00)	7.04 (5.96, 9.25)	0.4
Creatinine	0.80 (0.68, 0.97)	0.80 (0.60, 0.96)	0.80 (0.71, 1.00)	0.2
*Reason for labeling as BR-PDAC*^*&*^				
Anatomic only	57 (35%)	54 (49%)	3 (6%)	< 0.001
Biologic only	68 (42%)	21 (19%)	47 (94%)	< 0.001
Both anatomic and biologic	30 (19%)	30 (27%)	0	< 0.001
Functional status	5 (3%)	5 (4.5%)	0	0.29

^1^Median (IQR); *n* (%)

^2^Wilcoxon rank sum test; Pearson’s chi-squaredd test; Fisher’s exact test; Wilcoxon rank sum exact test

^&^The reason for labeling one patient who was referred to NT as BR-PDAC could not be found

*HTN* hypertension, *IHD* ischemic heart disease, *COPD* chronic obstructive pulmonary disease, *CVA* cerebrovascular accident, *CRF* chronic renal failure, *CBD* common bile duct, *CEA* carcinoembryonic antigen, *WBC* white blood cells

Among patients who underwent NT followed by surgery, 66 (83%) underwent pancreaticoduodenectomy (PD), including 6 who required total pancreatectomy (TP), while 13 (17%) underwent left pancreatectomy with splenectomy. In comparison, within the upfront surgery group, 45 patients (90%) underwent PD and 5 (10%) underwent left pancreatectomy with splenectomy. Owing to sample size constraints, subsequent analyses focused exclusively on patients who underwent PD and TP. Patients in the NT group were more likely to require vascular reconstruction during surgery but exhibited lower rates of pancreatic fistula formation compared with the upfront surgery group. Additionally, final pathology findings in the NT group demonstrated smaller tumor sizes and lower rates of lymphovascular invasion (Table 2). Other operative and postoperative outcomes were comparable between the groups (Table 2).

**Table 2:** Postoperative characteristics of patients with BR-PDAC who underwent PD with or without NT

Characteristic	Total, *n* = 111^1^	Upfront surgery, *n* = 45^1^	Neoadjuvant + surgery, *n* = 66^1^	*p*-value^2^
Vessel reconstruction	29 (26%)	1 (2.2%)	28 (42%)	< 0.001
Abnormal bleeding during surgery	16 (14%)	7 (18%)	9 (15%)	0.78
Length of stay from surgery in days	11 (9, 15)	12 (8, 16)	11 (9, 16)	0.35
SSI	11 (9%)	2 (4.5%)	9 (14%)	0.12
Pancreatic fistula	16 (14%)	10 (24%)	6 (9%)	0.05
Pancreatic fistula ≥ grade 2	7 (6%)	3 (7.3%)	4 (6.3%)	> 0.9
Clavien–Dindo > 3a	14 (13%)	6 (19%)	8 (19%)	0.43
Clavien–Dindo < 2	55 (49%)	25 (55%)	33 (50%)	0.55
Reoperation	12 (11%)	3 (7.0%)	9 (14%)	0.35
Readmission	35 (31%)	15 (36%)	20 (31%)	0.67
Tumor size (cm)	3.00 (2.00, 3.80)	3.20 (2.50, 4.50)	2.50 (2.00, 3.50)	0.006
Perineural invasion	62 (56%)	25 (61%)	37 (60%)	>0.9
Lymphovascular invasion	25 (22%)	16 (39%)	9 (15%)	0.009
Margins of resection_R1	36 (32%)	11 (24%)	25 (38%)	0.19

^1^Median (IQR); n (%)

^2^Wilcoxon rank sum test; Pearson’s chi-squared test; Fisher’s exact test; Wilcoxon rank sum exact test

*SSI* surgical site infection

**Fig. 1:**
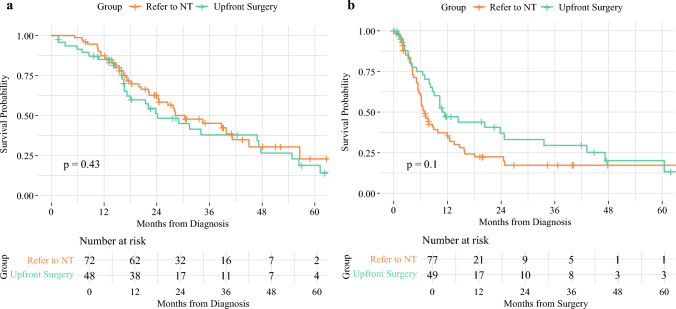
Long-term outcomes in patients with BR-PDAC who underwent surgery with and without NT: **a** OS from diagnosis and **b** DFS from surgery

The proportion of patients who received adjuvant therapy after surgery was similar between those who underwent NT and those who proceeded directly to upfront surgery (61% versus 66%, respectively; *p *= 0.31). However, patients in the upfront surgery group-initiated adjuvant chemotherapy significantly earlier, with a mean interval of 66.48 ± 24.21 days post-surgery compared with 81.78 ± 36.38 days in the NT group (*p *= 0.04). In the entire group of patients with BR-PDAC who underwent surgery, tumor size, and positive surgical margins were associated with early recurrence on univariate analysis and tumor size remained statistically significant on multivariate analysis as well (Supplementary Table S1). We could not find factors that were significantly associated with mortality on univariate or multivariate analysis (Supplementary Table S2). No significant difference in overall survival (OS) or disease-free survival (DFS) was observed between patients with BR-PDAC who underwent surgery with or without NT (Fig. 1 and Supplementary Tables S1 and S2).

**Fig. 2:**
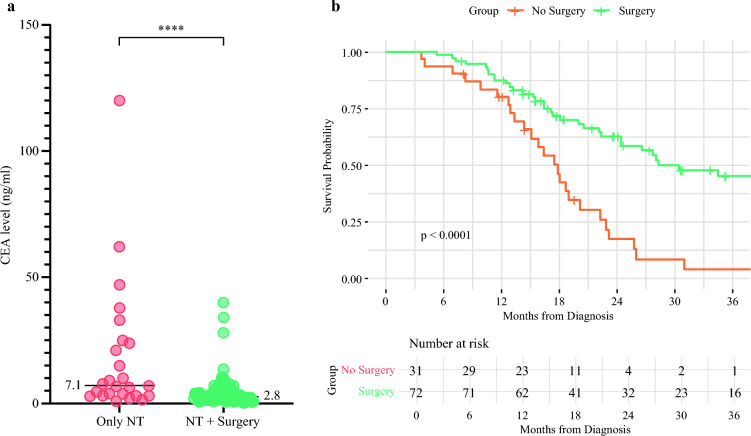
CEA levels and long-term survival in patients referred for NT with and without surgery. **a** Scatter plot comparing CEA levels (ng/ml) between patients with BR-PDAC who were referred to NT with and without surgery. Each dot represents an individual patient. Median CEA levels are indicated below each group (7.1 ng/ml for Only NT and 2.8 ng/ml for NT + Surgery). **** Statistical significance (*p *< 0.0001). **b** Long-term outcomes in patients with BR-PDAC who were referred to NT with and without surgery

We further analyzed the subset of patients with BR-PDAC who were referred for NT. Among the 111 patients, 78 (70%) proceeded to surgical resection while 33 (30%) did not undergo tumor resection surgery. The time between diagnosis and initiation of NT was comparable between those who reached surgery and those who did not (44 ± 35 days versus 51 ± 27 days in those who reached surgery and those who did not, respectively; *p = *0.14). The perioperative characteristics of these groups are detailed in Table 3. Of the entire cohort referred for NT, 81 patients (73%) received FOLFIRINOX as the primary treatment, 10 (9%) received a gemcitabine-based regimen, and 9 (8%) were treated with FOLFOX. Alternative protocols, including FOLFIRI, XELOX, or checkpoint inhibitors, were used in six patients (5%). Additionally, 24 patients (22%) underwent neoadjuvant external beam radiation therapy. No significant difference was observed in the percentage of patients who received FOLFIRINOX between the two groups (*p *= 0.23). At the decision point for surgery, patients who proceeded to surgical resection received an average of 6 ± 2.22 cycles of chemotherapy, and those who did not undergo surgery received 6 ± 2.96 cycles (*p *= 0.29). Notably, among patients for whom surgery was deemed unfeasible, many transitioned to second-line therapy and subsequently received additional cycles of chemotherapy. The primary reasons for failure to proceed to surgery in the NT group were local tumor progression, which rendered resection unfeasible in 11 patients (39%), and the development of new metastases following NT, in 6 patients (18%). Additionally, nine patients (27%) were taken to surgery but had their procedures aborted owing to intraoperative findings. Among these, five patients had newly discovered metastases, and four patients were found to have locally advanced tumors. Patients with BR-PDAC who did not proceed to surgical resection had higher serum carcinoembryonic antigen (CEA) levels at diagnosis compared with those who underwent surgery. Among the 33 patients who eventually did not proceed to surgery, 15 (45%) had elevated CEA levels at diagnosis (above 5 ng/ml) compared with 17 (22%) in those who were eventually operated on (Table 3). Additionally, they exhibited elevated white blood cell (WBC) counts and bilirubin levels. These patients also had higher rates of CBD stent placement after diagnosis and were more likely to receive neoadjuvant radiation therapy. Univariate logistic regression analysis demonstrated that higher CEA levels at diagnosis were significantly associated with reduced odds of undergoing surgery after NT (OR 0.93; 95% CI 0.88–0.97, *p *= 0.003). After adjusting for albumin and bilirubin, elevated CEA remained the only significant predictor, with a 5-unit increase reducing the odds of undergoing surgery by 32%. The C-statistic of the model was 0.75 (95% CI 0.63–0.87), which implies a good discriminative ability of CEA for those who will undergo surgery. The distribution of CEA levels of patients who failed to reach surgery compared with those who were operated on after NT is depicted in Fig. 2a.

**Table 3:** Characteristics of patients with BR-PDAC referred to NT who reached or could not reach tumor resection

Characteristic	All, *n* = 111^1^	Only NT, *n* = 33^1^	NT + surgery, *n* = 78^1^	*p*-value^2^
*Demographics and medical history*				
Age (years)	67 (60, 73)	68 (59, 79)	67 (60, 73)	0.3
Gender female	50 (45%)	11 (33%)	39 (49%)	0.15
Active smoker	40 (36%)	8 (24%)	32 (41%)	0.2
Diabetes	53 (48%)	12 (36%)	41 (52%)	0.33
HTN	43 (39%)	9 (27%)	34 (43%)	0.4
IHD	16 (14%)	3 (9%)	12 (15%)	0.51
COPD	1 (1%)	0	1 (1.3%)	0.54
Asthma	4 (4%)	0	4 (5.1%)	0.21
CVA	4 (4%)	3 (9%)	1 (1.3%)	0.02
CRF	3 (3%)	1 (3%)	2 (2.5%)	0.79
*Presenting symptoms*				
Pruritus	4 (4%)	2 (6%)	2 (2.6%)	0.3
Abdominal pain	52 (47%)	14 (42%)	38 (49%)	> 0.9
Weight loss	48 (43%)	12 (36%)	36 (47%)	0.5
Back pain	13 (12%)	2 (6%)	11 (14%)	0.3
Jaundice	39 (35%)	14 (42%)	25 (32%)	0.13
No presenting symptoms	7 (6%)	0	7 (9.1%)	0.09
Other presenting symptoms	20 (18%)	5 (15%)	15 (19%)	0.79
CBD stenting	46 (41%)	15 (45%)	31 (40%)	0.08
*Relevant laboratory indices at disease presentation*				
CA19-9	312 (84, 1,306)	459.5 (153.0, 2904.0)	274.0 (76.0, 1000.0)	0.119
CEA	3 (2, 7)	7.10 (3.20, 25.00)	2.90 (1.70, 4.90)	< 0.001
CEA > 5 ng/ml	40 (36%)	15 (45%)	17 (22%)	0.02
Albumin	3.80 (3.50, 4.10)	3.88 (3.61, 4.15)	3.72 (3.12, 4.32)	0.096
Bilirubin	0.5 (0.39, 1.0)	3.83 (0.5, 9.48)	1.16 (0.4, 3.46)	0.04
WBC	6.95 (5.66, 9.00)	8.57 (5.7, 11.44)	7.14 (4.7, 9.87)	0.04
Creatinine	0.80 (0.60, 0.96)	0.80 (0.64, 1.00)	0.80 (0.60, 0.91)	0.92
No. of chemo cycles	6.00 (4.00, 7.00)	6.00 (4.00, 8.00)	6.00 (4.00, 7.00)	0.29
Received FOLFIRINOX	83 (74%)	22 (66%)	61 (78%)	0.23
Chemo side-effects	47 (42%)	11 (33%)	36 (47%)	0.77
Radiation	42 (38%)	15 (45%)	7 (34%)	<0.001

^1^Median (IQR); *n* (%)

^2^Wilcoxon rank sum test; Pearson’s chi-squared test; Fisher’s exact test

*HTN* hypertension, *IHD* ischemic heart disease, *COPD* chronic obstructive pulmonary disease, *CVA* cerebrovascular accident, *CRF* chronic renal failure, *CBD* common bile duct, *CEA* carcinoembryonic antigen, *WBC* white blood cells

The most common reason for not undergoing surgery in the patients with elevated CEA at presentation was local tumor progression, identified either on imaging or during surgery, accounting for 60% of cases. The development of distant or peritoneal metastases, detected through imaging or intraoperative assessment, was the second most frequent cause, occurring in 34% of patients. Additionally, 6% of patients were deemed unfit for surgery owing to deconditioning following NT. Of note, none of the patients with elevated CEA who failed to reach surgery owing to local tumor progression developed metastases during at least 6 months from starting NT. Of those patients with BR-PDAC who proceeded to surgical resection, 48 (61%) were able to receive adjuvant therapy. The mean number of adjuvant chemotherapy cycles was 4.12 ± 3.21. Notably, only 43% of patients with BR-PDAC referred for NT were able to complete the full treatment plan, including NT, surgery, and adjuvant therapy. Patients who underwent surgery had significantly improved OS compared with those who did not proceed to surgery, as depicted in Fig. 2b.

We subsequently analyzed a separate cohort of patients diagnosed with resectable PDAC who underwent PD at our center during the same period. These patients were compared with those who had BR-PDAC and underwent PD after NT as well as those with BR-PDAC who proceeded directly to upfront surgery (Table 4). Among the three groups, patients with BR-PDAC who received NT were the youngest, required more frequent vascular resections during surgery, and had the lowest rates of postoperative pancreatic fistula formation. Additionally, their tumors were the smallest in size and demonstrated the lowest rates of lymphovascular invasion on final pathology (Table 4).

**Table 4:** Postoperative results of patients with BR-PDAC with and without NT who underwent PD to patients with resectable PDAC who underwent PD

	Total, *n* = 265^1^	Resectable, *n* = 154^1^	Borderline, upfront surgery, *n* = 45^1^	Borderline, NT + surgery, *n* = 66^1^	*p*-value^2^
Age (years)	69.94 (62.53, 75.64)	69.65 (62.6, 75)	69.0 (62.9, 75.9)	66.9 (60.2, 72.8)	0.006
Gender female	126 (47%)	75 (48%)	18 (40%)	33 (50%)	0.53
Vessel reconstruction	43 (16%)	14 (9%)	1 (2%)	28 (42%)	<0.0001
Abnormal bleeding during surgery	31 (12%)	15 (10%)	7 (18%)	9 (15%)	0.27
Length of stay from surgery in days	18	11.6 (8.6, 18)	11.5 (8.0, 15.5)	10.9 (8.7, 15.8)	0.63
SSI	34 (13%)	23 (15%)	2 (4%)	9 (14%)	0.22
Pancreatic fistula	59 (22%)	43 (28%)	10 (24%)	6 (9%)	0.01
Pancreatic fistula ≥ grade 2	27 (10%)	20 (13%)	3 (7%)	4 (6%)	0.25
Clavien–Dindo > 3a	44 (17%)	26 (17%)	9 (21%)	9 (14%)	0.63
Clavien–Dindo <2	159 (60%)	101 (65%)	25 (59%)	33 (52%)	0.18
Readmission	99 (37%)	64 (41%)	15 (35%)	20 (31%)	0.29
Reoperation	37 (14%)	25 (16%)	3 (7%)	9 (14%)	0.30
Tumor size in cm	2.5 (2.00, 3.5)	2.5 (2.0, 3.5)	3.2 (2.3, 4.5)	2.5 (2.0, 3.5)	0.003
Perineural invasion	158 (59%)	96 (62%)	25 (61%)	37 (60%)	0.85
Lymphovascular invasion	76 (29%)	51 (33%)	16 (39%)	9 (14%)	0.008
Margins of resection_R1	85 (32%)	49 (32%)	11 (26%)	25 (40%)	0.10

^1^Median (IQR); *n* (%)

^2^Wilcoxon rank sum test; Pearson’s chi-squared test; Fisher’s exact test; Wilcoxon rank sum exact test

*SSI* surgical site infection

The rate of patients with resectable tumors who received adjuvant therapy was 67% (104 patients), compared with 64% (29 patients) in those with BR-PDAC who underwent upfront surgery and 57% (38 patients) in those with BR-PDAC operated on after NT (*p *= 0.37).

**Fig. 3.**
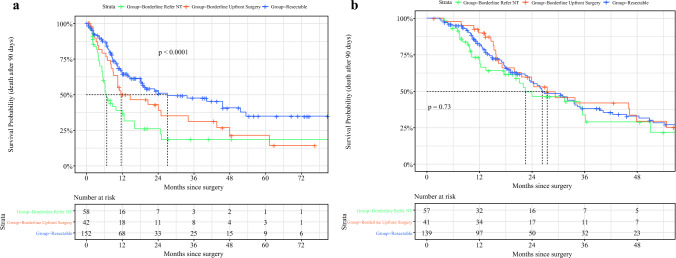
Long-term outcomes in patients with resectable PDAC compared with patients with borderline PDAC who underwent surgery with and without NT: **a** DFS from surgery and **b** OS from diagnosis

DFS was significantly longer in the resectable group, followed by those with BR-PDAC who underwent upfront surgery (*p *< 0.0001). Median survival time for DFS was 27 months in the resectable group, 11.6 months in the BR-PDAC upfront surgery group, and 6.7 months in the BR-PDAC group referred for NT (Fig. 3a). In contrast, no significant difference in OS was observed between the groups (*p *= 0.73) (Fig. 3b). However, median overall survival highlighted a notable difference: patients in the NT group had the shortest median survival time of 22.5 months, followed by the resectable group of 26.4 months, with the group of patients with BR-PDAC with upfront surgery showing the longest median survival time of 27.5 months.

## Discussion

In this study, we analyzed a cohort of patients with BR-PDAC to assess the outcomes of various treatment strategies. Specifically, we investigated factors associated with failure to achieve surgical resection in patients referred for NT. We also evaluated the long-term outcomes of those who underwent surgical resection, comparing them with patients who did not proceed to surgery and those who underwent surgery for resectable disease. This report reflects a “real-life” experience from a large oncology and surgical referral center, highlighting that decision-making regarding treatment options for patients with BR-PDAC can sometimes diverge from consensus guidelines.

For example, in our cohort, anatomical criteria for borderline resectability predominantly guided the decision to refer patients to NT, whereas those classified as having BR-PDAC on the basis of biological factors—such as elevated tumor markers or peripancreatic lymphadenopathy—were more often directed to upfront surgery. Notably, 94% of patients who underwent upfront resection met BR criteria owing to biological rather than anatomical factors. This practice may explain the seemingly paradoxical finding that CA19-9 levels at presentation were higher in patients referred for upfront surgery compared with those directed to NT.

The inherent differences in major disease characteristics between patients with BR-PDAC referred to upfront surgery and those referred to NT complicate the assessment of NT’s impact on long-term outcomes. Nevertheless, our findings suggest that, despite an initial anatomical disadvantage, patients referred to NT who eventually underwent surgery demonstrated smaller tumors with lower rates of lymphovascular involvement compared with those taken directly to surgery. However, no significant differences in DFS or OS were observed between the groups.

It is plausible that these findings underscore the benefits of NT. By addressing the anatomical challenges posed by the tumor, NT may have equalized long-term outcomes between patients with BR-PDAC owing to anatomical criteria and those classified on the basis of biological criteria. This suggests that NT might help mitigate the adverse effects of unfavorable tumor anatomy, allowing these patients to achieve outcomes comparable to those with biologically defined BR-PDAC. However, despite this favorable initial response to NT as reflected by pathology characteristics post-surgery, the OS and DFS were not improved in comparison with the other patient cohorts.

We found that elevated CEA levels at presentation were a significant marker for failure to achieve surgical resection after NT. Serum CEA was shown to be a marker for disease progression and a prognostic factor for long-term outcomes in patients with PDAC.^10^ Kato et al. recently demonstrated that pre-treatment CEA levels were the most significant prognostic factor in patients with PDAC who were referred to NT.^11^ Doppenberg et al. showed that, in patients with localized PDAC who were referred to NT and had non-elevated CA19-9 levels, high CEA at baseline and during restaging was the only predictor for worse overall survival.^12^ While these and other studies primarily focused on long-term oncologic outcomes such as DFS and OS in patients who underwent surgery, our study provides a novel perspective by evaluating CEA as a marker for a different outcome—failure to reach surgery after NT. This distinction is clinically significant, as it may help refine treatment strategies by identifying patients with BR-PDAC who might benefit more from upfront surgery rather than NT. Moreover, the aforementioned studies did not specify the pattern of recurrence in those with elevated CEA. Notably, in our cohort, most patients with elevated CEA failed to proceed to surgery owing to local tumor progression rather than disseminated disease. Although CA 19-9 has been widely recognized as a marker associated with metastatic disease in pancreatic cancer,^13^ the prognostic role of CEA in PDAC, particularly regarding its association with metastatic disease versus local progression, is less well established. Importantly, none of the patients with elevated CEA who failed to reach surgery owing to local progression developed metastases during at least 6 months from starting NT. This observation may suggest that, in these patients, the “window of opportunity” for surgery could be missed if surgery is delayed for NT. Therefore, we suggest that upfront surgery should be considered for patients with localized BR-PDAC who present with elevated CEA levels. This notion, of course, warrants validation via larger and prospective studies.

Unsurprisingly, patients with BR-PDAC who were referred to NT and successfully underwent surgery had significantly better long-term outcomes compared with those who did not reach surgery. However, these patients still experienced shorter DFS compared with those with resectable tumors who underwent surgery, despite comparable tumor sizes between the groups and lower rates of lymphovascular involvement in the patients with BR-PDAC after NT.

This study has several limitations, primarily stemming from its retrospective design and the relatively small sample size, being based on the experience of a single center. Additionally, reliance on electronic medical records and incomplete documentation may have impacted the accuracy of some statistical conclusions. However, regarding predictors of failure to proceed to surgery, the outcomes are definitive (surgery performed or not), and the groups are delineated. This clarity allows for a more reliable evaluation of objective predictors, such as laboratory indices at the time of diagnosis.

In conclusion, this study highlights the importance of tailored treatment strategies for patients with BR-PDAC. Elevated CEA levels were a significant predictor of failure to reach surgery after NT, mainly owing to local progression. This raises the notion that upfront surgery may be preferable in these cases. While NT improved tumor characteristics in surgical candidates, no differences in DFS or OS were observed between NT and upfront surgery groups. Despite its limitations, this study underscores the need for individualized approaches and further research to optimize outcomes in BR-PDAC.

## Supplementary Information

Below is the link to the electronic supplementary material.

Supplementary file1 (DOCX 20 KB)
