# Supplementary material for: Elevated Carcinoembryonic Antigen Levels Predict Failure to Reach Surgery in Patients with Borderline Resectable Pancreatic Cancer Referred to Neoadjuvant Therapy
Source: Ann Surg Oncol. 2025 May 13;32(9):6501–10. doi: 10.1245/s10434-025-17433-3 (PMC12317868; doi:10.1245/s10434-025-17433-3)
Supplement: Supplementary file 1 — Supplementary file1 (DOCX 20 KB) [file 10434_2025_17433_MOESM1_ESM.docx]

**Table S1.** Univariate and multivariate analyses to assess factors associated with disease recurrence in patients who underwent PD with or without NT.

| **Variable** | **Univariate_OR** | **P-value_**  **Univariate** | **Multivariate_OR** | **P-value_**  **Multivariate** |
| --- | --- | --- | --- | --- |
| Upfront Surgery | 1.03 (0.47 - 2.27) | 0.942 | 1.22 (0.43 - 3.55) | 0.711 |
| Age | 0.97 (0.93 - 1.02) | 0.242 |  |  |
| CBD stenting | 1.59 (0.73 - 3.54) | 0.246 |  |  |
| CA19-9 | 1.00 (1.00 - 1.00) | 0.137 | 1.00 (1.00 - 1.00) | 0.251 |
| CEA | 1.01 (0.98 - 1.08) | 0.516 | 1.02 (0.98 - 1.09) | 0.537 |
| Bilirubin | 1.00 (0.92 - 1.09) | 0.989 | 0.96 (0.86 - 1.07) | 0.469 |
| Vessel reconstruction | 1.01 (0.43 - 2.48) | 0.974 |  |  |
| Pancreatic fistula | 2.09 (0.67 - 7.95) | 0.230 |  |  |
| Lymphovascular invasion | 1.79 (0.69 - 5.06) | 0.246 | 1.54 (0.52 - 4.94) | 0.450 |
| Tumor size in cm | 1.47 (1.07 - 2.11) | 0.024 |  |  |
| Margins of resection_R1 | 2.95 (1.22 - 7.75) | 0.021 | 3.07 (1.19 - 8.54) | 0.024 |

| **Variable** | **Univariate_OR** | **P-value_**  **Univariate** | **Multivariate_OR** | **P-value_**  **Multivariate** |
| --- | --- | --- | --- | --- |
| Upfront Surgery | 0.68 (0.31 - 1.49) | 0.343 | 0.65 (0.22 - 1.92) | 0.441 |
| Age | 0.97 (0.93 - 1.01) | 0.121 |  |  |
| CBD stenting | 0.74 (0.34 - 1.61) | 0.455 |  |  |
| CA19-9 | 1.00 (1.00 - 1.00) | 0.282 | 1.00 (1.00 - 1.00) | 0.217 |
| CEA | 1.05 (1.00 - 1.14) | 0.177 | 1.08 (1.01 - 1.20) | 0.118 |
| Bilirubin | 0.93 (0.84 - 1.02) | 0.178 | 0.99 (0.87 - 1.10) | 0.805 |
| Vessel reconstruction | 1.24 (0.52 - 2.93) | 0.617 |  |  |
| Pancreatic fistula | 1.17 (0.39 - 3.41) | 0.778 |  |  |
| Lymphovascular invasion | 0.37 (0.12 - 0.98) | 0.055 | 0.39 (0.10 - 1.21) | 0.120 |
| Tumor size in cm | 0.91 (0.67 - 1.22) | 0.538 |  |  |
| Margins of resection_R1 | 0.46 (0.19 - 1.07) | 0.077 | 0.52 (0.19 - 1.33) | 0.177 |

**Table S2.** Univariate and multivariate analyses to assess factors associated with disease survival in patients who underwent PD with or without NT.
